# A Combined Model of Human iPSC‐Derived Liver Organoids and Hepatocytes Reveals Ferroptosis in DGUOK Mutant mtDNA Depletion Syndrome

**DOI:** 10.1002/advs.202004680

**Published:** 2021-03-08

**Authors:** Jingyi Guo, Lifan Duan, Xueying He, Shengbiao Li, Yi Wu, Ge Xiang, Feixiang Bao, Liang Yang, Hongyan Shi, Mi Gao, Lingjun Zheng, Huili Hu, Xingguo Liu

**Affiliations:** ^1^ University of Science and Technology of China Bioland Laboratory (Guangzhou Regenerative Medicine and Health Guangdong Laboratory) Joint School of Life Sciences Guangzhou Institutes of Biomedicine and Health Chinese Academy of Sciences Guangzhou Medical University Hefei 230026 China; ^2^ CAS Key Laboratory of Regenerative Biology Guangdong Provincial Key Laboratory of Stem Cell and Regenerative Medicine Institute for Stem Cell and Regeneration Guangzhou Institutes of Biomedicine and Health University of Chinese Academy of Sciences Chinese Academy of Sciences Guangzhou 510530 China; ^3^ The Key Laboratory of Experimental Teratology Ministry of Education and Department of Genetics School of Basic Medical Sciences Shandong University Jinan 250012 China

**Keywords:** ferroptosis, induced pluripotent stem cells, mitochondria, mitochondrial DNA, N‐acetylcysteine

## Abstract

Mitochondrial DNA depletion syndrome (MDS) is a group of severe inherited disorders caused by mutations in genes, such as deoxyribonucleoside kinase (DGUOK). A great majority of DGUOK mutant MDS patients develop iron overload progressing to severe liver failure. However, the pathological mechanisms connecting iron overload and hepatic damage remains uncovered. Here, two patients’ skin fibroblasts are reprogrammed to induced pluripotent stem cells (iPSCs) and then corrected by CRISPR/Cas9. Patient‐specific iPSCs and corrected iPSCs‐derived high purity hepatocyte organoids (iHep‐Orgs) and hepatocyte‐like cells (iHep) are generated as cellular models for studying hepatic pathology. DGUOK mutant iHep and iHep‐Orgs, but not control and corrected one, are more sensitive to iron overload‐induced ferroptosis, which can be rescued by N‐Acetylcysteine (NAC). Mechanically, this ferroptosis is a process mediated by nuclear receptor co‐activator 4 (NCOA4)‐dependent degradation of ferritin in lysosome and cellular labile iron release. This study reveals the underlying pathological mechanisms and the viable therapeutic strategies of this syndrome, and is the first pure iHep‐Orgs model in hereditary liver diseases.

## Graphical abstract

Iron overload is an important feature in deoxyribonucleoside kinase mutant mitochondrial DNA depletion syndrome. A combined model of patient‐specific induced pluripotent stem cells‐derived liver organoids and hepatocytes reveals a sensitivity to iron overload‐induced ferroptosis in patients. This ferroptosis is a process by NCOA4‐dependent degradation of ferritin in lysosome and cellular labile iron release.

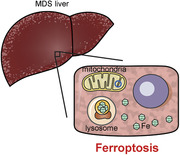

## 1 Introduction

Mitochondrial DNA depletion syndrome (MDS) is a group of severe, phenotypically heterogeneous, recessively inherited disorders characterized by marked reduction of the mtDNA content in affected tissues and organs.^[^ ^1^ ^]^ Deoxyribonucleoside kinase (DGUOK) mutation is the major causes of hepatocerebral form MDS, accounting for ≈20% of all MDS cases, and is most common genetic factor of hepatic MDS.^[^ ^2^ ^]^ DGUOK mediates the phosphorylation of deoxyguanosine and deoxyadenosinepurine into the corresponding nucleotides in mitochondria. *DGUOK* mutation unbalance the mitochondrial deoxyribonucleoside triphosphate (dNTP) pool which can cause mtDNA synthesis breakdown, eventually cause MDS.^[^ ^3^ ^]^ The significant clinical presentation of *DGUOK* mutant MDS patients is liver impairment, or accompanied with epilepsy, hypotonia, ataxia, and nystagmus. Patients usually died early due to severe liver failure before 2 years’ old. There are no effective therapies for this disease, expect liver transplantation, leading to poor prognosis in the majority of patients. Therefore, finding effective therapeutic targets of pathological processes of the liver failure is brook no delay.

Some research efforts have been made focusing on the dNTPs pools using fibroblasts or myoblasts of patients, but they are insufficient to uncover mechanisms underlying the severe liver failure. On the basis of clinical case studies, almost all *DGUOK* mutant patients developed iron overload progressing to liver failure.^[^ ^4^, ^5^, ^6^ ^]^ The pathological mechanism connecting iron overload and liver failure in DGUOK mutant MDS, however, remains to be uncovered.

Liver is a major site of iron storage and plays a central role in systemic iron homeostasis. This makes liver a preferential target of iron overload toxicity.^[^ ^7^ ^]^ Alcoholic liver disease, nonalcoholic fatty liver disease, acetaminophen overdose‐induced acute liver failure, and viral hepatitis are all related to liver iron loading. Excess iron‐induced oxidative stress is thought to give rise to these diseases.^[^ ^8^ ^]^ In recent years, an iron‐dependent form of cell death, known as ferroptosis, has been widely investigated. During the process of ferroptosis, the redox‐active ability of iron produce free radicals, leading to lipid peroxidation and initiation of signaling pathways crucial for cell death.^[^ ^9^ ^]^ Inhibition of system X_c_^−^, glutathione (GSH) or cysteine depletion, and glutaminolysis can cause ferroptosis.^[^ ^10^, ^11^, ^12^ ^]^ Glutathione peroxidase 4 (GPX4) and ferroptosis suppressor protein 1, as key components in elimination lipid peroxides, can protect cell from ferroptosis.^[^ ^13^, ^14^ ^]^ Previous studies revealed that ferroptosis is also an autophagic process with degradation of ferritin to release iron to trigger ferroptosis.^[^ ^15^, ^16^ ^]^ Ferroptosis is an important factor in many diseases, like hemochromatosis,^[^ ^17^ ^]^ liver toxicity,^[^ ^18^ ^]^ liver fibrosis,^[^ ^19^ ^]^ cardiomyopathy,^[^ ^20^, ^21^ ^]^ acute renal failure,^[^ ^22^ ^]^ and neurodegenerative diseases.^[^ ^23^ ^]^ While iron overload in liver is a characteristic clinical feature of DGUOK mutant patients, directly relevant research is not yet reported.

It is critical to establish a powerful model system mimicking the liver of DGUOK mutant MDS enabling the studies of intrinsic mechanism, as well as drug discovery for clinical therapeutics. It has been difficult to acquire liver samples from DGUOK mutant patients for research, while either fibroblasts from patients or animal models exist different features with human biology. The breakthrough in the generation of induced pluripotent stem cells (iPSCs),^[^ ^24^ ^]^ iPSCs derived 2D hepatocyte like cells^[^ ^25^ ^]^ and advances in 3D cell culture^[^ ^26^ ^]^ represents a promising approach whereby human iPSCs can provide a renewable source of hepatocytes and liver organoids which carry the whole genetic background of a patient. iPSCs‐derived 2D hepatocyte‐like cells (iHep) is a good model. Many studies used iHep to model liver diseases and proved its efficacy in exploring the pathological mechanisms of a broad range of diseases. A good example is the work in the first toxicity iHep model in a genetic disease of valproic acid induced hepatotoxicity in Alpers syndrome.^[^ ^27^ ^]^ Another advance technology of in vitro model is the generation of organoids. The 3D organoids system provide the advantage in maintaining the cell‐to‐cell contacts and the 3D spatial cellular organization of tissues or organs. Recently, a long‐term expansion of functional 3D liver organoids was established with the advantage of pure hepatocyte‐organoids with a low quantity of cholangiocyte‐like cells.^[^ ^26^ ^]^ This 3D liver organoids system was used in some liver disease like alcoholic liver injury,^[^ ^28^ ^]^ hepatocellular carcinomas,^[^ ^29^ ^]^ but not be used in genetic liver disease yet. Moreover, human iPSCs can be genetically edited by the CRISPR/Cas9 system to supply isogenic controls.

Here, we establish an in vitro liver disease model of liver organoids and hepatocytes developed from iPSCs of DGUOK mutant patients, along with associated controls and isogenic cell lines corrected by the CRISPR/Cas9 system. Using this model, we show that mtDNA depletion and respiration dysfunction in the patient iPSCs‐derived hepatocyte‐like cells and both iHep and iHep‐generated organoids (iHep‐Orgs) are more sensitive to iron overload‐induced ferroptosis which could recused by the GSH precursor, N‐acetylcysteine (NAC). This ferroptosis is a process mediated by nuclear receptor co‐activator 4 (NCOA4)‐dependent degradation of ferritin in lysosome. Our study provides the first hereditary liver disease model using pure hepatocyte organoids, giving critical mechanistic insight into liver failure of DGUOK mutant MDS.

## 2 Results

### 2.1 Generation of Liver Organoids and Hepatocytes Model from CRISPR/cas9‐mediated Gene Corrected DGUOK Mutant iPSCs

**Figure 1:**
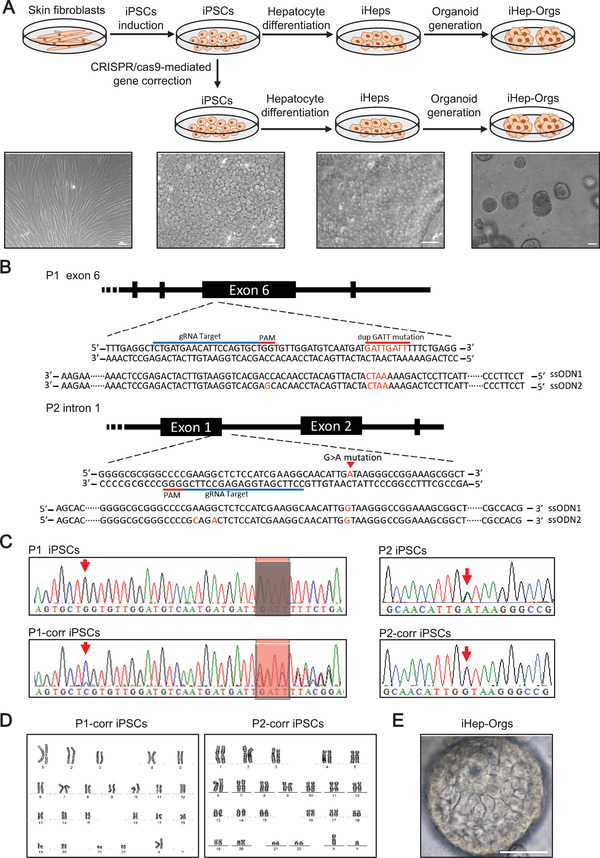
Generation of hepatocytes and organoids model from CRISPR‐cas9‐mediated gene corrected *DGUOK* mutant iPSCs. A) Schematic representation of iPSCs induction, gene correction, hepatocyte differentiation, and organoid generation. Scale bar, 50 µm. B) Experimental design of site‐specific gRNA and ssODN for CRISPR/cas9. C) Sequencing results of the DGUOK mutation sites before and after gene correction. D) Karyotype of iPSC clones after gene correction. E) Images of iHep‐Orgs. Scale bar, 50 µm.

Skin fibroblasts from two patients with different mutation sites in *DGUOK* ^[^ ^30^ ^]^ were reprogrammed to iPSCs. As the diagram in **Figure** **1**A, we derived iPSCs to generate liver organoids and hepatocytes. The reprogrammed iPSCs displayed pluripotent characteristics and normal karyotype (Figure S1A–C, Supporting Information). Patient 1, a girl, died at 2 months of age of liver failure with a homozygous mutation of 4 bp duplication in exon 6 (c.763 766dupGATT). Patient 2, a boy, died at 2 months of age of liver failure with compound heterozygosity consisting of three mutations: in exon 1(c.4G>T), intron 1 (c.142+1G>A), and exon 4 (c.591G>A) (Figure S1D, Supporting Information). The mutations A2S and c.591G>A came from his father and the mutation c.142+1G >A came from his mother.^[^ ^30^ ^]^ In order to correct the DGUOK gene, gRNAs were designed to target exon 6 of P1 and intron 1 of P2. Two ssODNs were designed for each patient as repair templates: one normal sequence, the other containing synonymous mutations to avoid the repaired allele being targeted and cut by the CRISPR system (Figure 1B). Then cas9 plasmid and single‐stranded oligodeoxynucleotides (ssODNs) were transfected into P1 and P2 iPSCs. After picking single clones, expanding and sequencing by PCR, we obtained *DGUOK* mutation corrected clones of two patients (Figure 1C). The karyotypes of the corrected iPSCs were also normal after gene editing (Figure 1D). Detection of possible off‐target sequences for CRISPR/cas9 showed there do not exist off‐target (Figure S2, Supporting Information).

Next, control iPSCs, patient iPSCs, and corrected iPSCs were differentiated into iHep as previously described.^[^ ^27^ ^]^ We examined the markers expressed in hepatic development and mature hepatocytes (Figure S3A,B, Supporting Information). These iHep also showed periodic acid‐Schiff (PAS) staining indicative of glycogen accumulation and indocyanine green (ICG) uptake and release indicative of hepatic excretory function (Figure S3C,D, Supporting Information). To generate liver organoids, iHep were seeded in Matrigel. About 2 weeks after seeding, iPSCs‐derived liver organoids (iHep‐Orgs) came out (Figure 1E), as H. Hu, et al. reported.^[^ ^26^ ^]^

### 2.2 Identification and Functional Characterization of iHep‐Orgs

**Figure 2:**
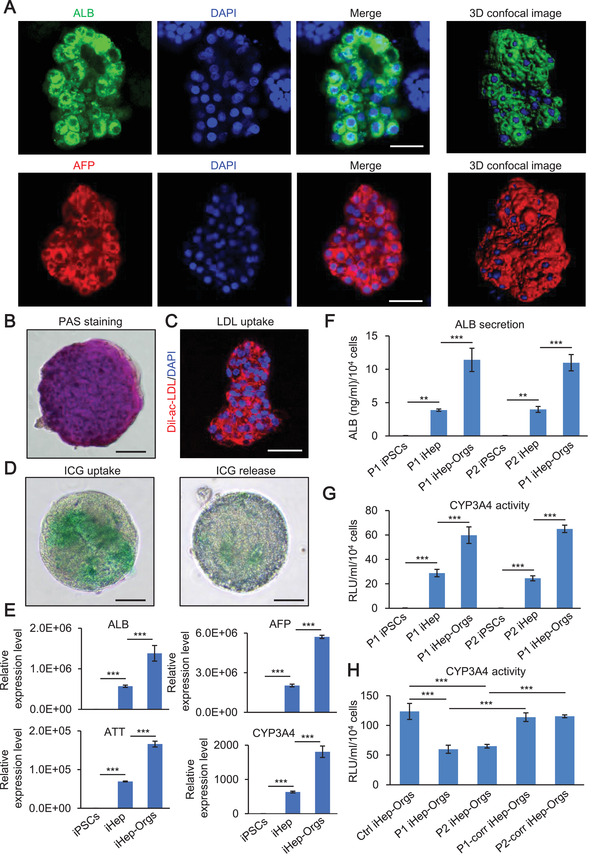
Characterization of iHep‐Orgs. A) Confocal images of iHep‐Orgs. ALB (green), AFP (red), and DAPI (blue). Scale bar, 50 µm. B) Glycogen accumulation evaluated by PAS staining in iHep‐Orgs. Scale bar, 100 µm. C) LDL uptake evaluated by Dil‐ac‐LDL fluorescent staining in iHep‐Orgs. Dil‐ac‐LDL (red) and DAPI (blue). Scale bar, 50 µm. D) ICG uptake and release in iHep‐Orgs. Scale bar, 100 µm. E) Relative expression level of liver specific marker in iPSCs, iHep, and iHep‐Orgs. F) Quantitation of ALB secretion in iPSCs, iHep, and iHep‐Orgs. G) Quantitation of CYP3A4 activity in iPSCs, iHep, and iHep‐Orgs. H) Quantitation of CYP3A4 activity in control, patient, and corrected iHep‐Orgs. These data are represented as mean ± SD (*n* = 3) and statistics were assessed using one‐way ANOVA followed by post‐hoc Holm–Sidak test, **p <0.01; ***p < 0.001.

We next performed the identification and functional characterization of iHep‐Orgs. Immunofluorescence showed that iHep‐Orgs expressed strong hepatocyte specific proteins: albumin (ALB) and fetal hepatic protein: fetoprotein (AFP) (**Figure** **2**A). Functionally, PAS staining and DiI‐Ac‐LDL indicated glycogen accumulation and low‐density lipoprotein (LDL) uptake in iHep‐Orgs (Figure 2B,C). We also observed ICG uptake and release in iHep‐Orgs (Figure 2D). The expression of hepatic genes (ALB, AFP, AAT, CYP3A4) was higher in iHep‐Orgs than iHep and iPSCs (Figure 2E). Then, analysis of ALB secretion and CYP3A4 activity suggested that iHep‐Orgs were more mature than iHep (Figure 2F,G). Moreover, we also detected ALB secretion and CYP3A4 activity in control, patient and corrected iHep‐Orgs. CYP3A4 activity declined in patient iHep‐Orgs (Figure 2H), but ALB secretion did not (Figure S3E, Supporting Information). Overall, these data indicate that iHep‐Orgs in 3D culture exhibited more functionally mature liver‐like characteristics than iHep in 2D culture, and patient iHep‐Orgs exhibited partial abnormal liver function compared with controls.

### 2.3 Mitochondrial DNA Depletion and Respiration Dysfunction in Patient iHep

**Figure 3:**
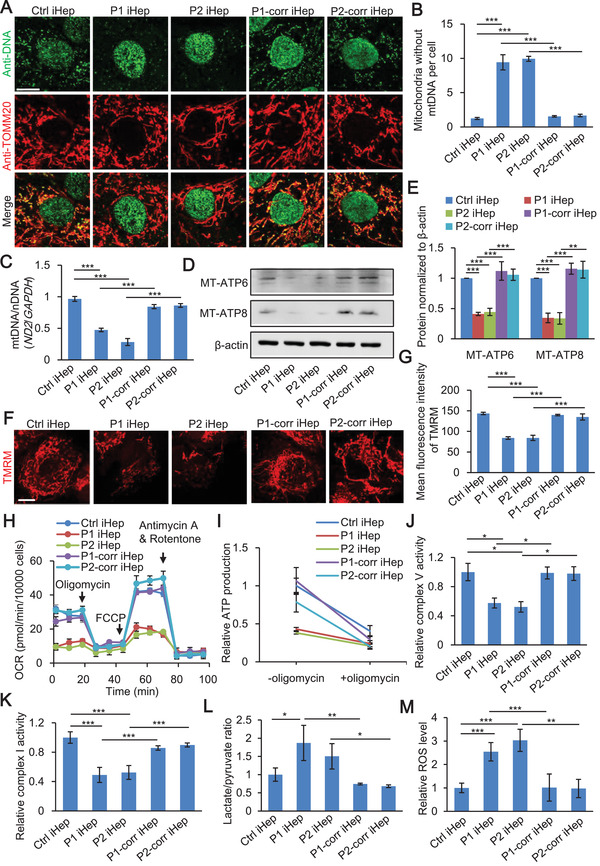
Mitochondria DNA depletion and respiration dysfunction in Patient iHep. A) Anti‐DNA and anti‐TOMM20 immunofluorescence in control iHep, patient iHep, and corrected iHep. Scale bar, 10 µm. B) Quantification of mitochondria without mtDNA in control, patient and corrected iHep (*n* = 3, 80 cells per group with three biological replicates). C) mtDNA content of control, patient and corrected iHep using the ratio of ND2 to GAPDH determining mtDNA/nDNA. D,E) Western blot of MT‐ATP6 and MT‐ATP8 level in control, patient and corrected iHep. F) Measurement of ΔΨ_m_ in control, patient and corrected iHep. Scale bar, 10 µm. G) Quantitation of mean fluorescence intensity of TMRM in control, patient, and corrected iHep (*n* = 3, 60 cells per group with three biological replicates). H) Oxygen consumption rate (OCR) measurements in control, patient and corrected iHep. I) Cellular ATP levels before or after oligomycin treatment in control, patient, and corrected iHep. J) Measurement of mitochondrial complex V in control, patient and corrected iHep. K) Measurement of mitochondrial complex I in control, patient and corrected iHep. L) Measurement of lactate/pyruvate ratio of control, patient and corrected iHep. M) Cellular ROS in control, patient and corrected iHep. These data are represented as mean ± SD (*n* = 3) and statistics was assessed using one‐way ANOVA followed by post‐hoc Holm–Sidak test, *p < 0.05;**p <0.01; ***p < 0.001.

DGUOK mutant patients are characterized by a severe reduction of the mtDNA content in liver.^[^ ^4^ ^]^ To assess mtDNA quantity, we performed immunofluorescence using an anti‐DNA and TOMM20 antibody in control, patient and corrected iHep. We showed that number of mitochondria without mtDNA per cell was much greater in patient iHep than in control and corrected iHep (**Figure** **3**A,B). We also measured the amount of mtDNA by quantitative PCR (qPCR) using an mtDNA‐encoded gene *ND2* and a nuclear gene, *GAPDH* (Figure 3C). These above results showed mtDNA depletion and a relative paucity of mtDNA nucleoids in DGUOK mutant patient iHep. Then we examined the transcript level of mtDNA and found mitochondrial mRNA levels were lower in patient iHep compared with those in DGUOK corrected iHep (Figure S4A, Supporting Information). We analyzed the translational level of mtDNA by measuring the expression of the mtDNA encoded proteins MT‐ATP6 and MT‐ATP8 by western blot. Levels of MT‐ATP6 and MT‐ATP8 were lower in patient iHep compared with those in control and DGUOK corrected iHep (Figure 3D,E). We also checked the quantity of mitochondria by detecting the mean fluorescence intensity of MitoTracker Deep Red and western blot of TOMM20, and showed that there was no difference among control, patient and corrected iHep (Figure S4B,C, Supporting Information). The morphology of mitochondria exhibited no significant difference among control, patient and corrected iHep (Figure S4D, Supporting Information). Together, these data indicate that mtDNA copy number and expression level but not mitochondrial quantity are decline in patient iHep.

To assess mitochondrial bioenergetics in control, patient and corrected iHep, we first measured ΔΨ_m_ by tetramethylrhodamine methyl ester (TMRM) staining. Patient iHep displayed decreased ΔΨ_m_ compared with control and corrected iHep (Figure 3F,G). We then measured oxygen consumption rate (OCR), an indicator of mitochondrial respiration and energy production, in control, patient and corrected iHep. We observed decreased basal oxygen consumption and diminished response to uncoupler (maximal respiration) in patient iHep compared with those in control and corrected iHep (Figure 3H; and Figure S4E, Supporting Information). Moreover, both cellular and mitochondrial ATP production was reduced in patient iHep relative to control and corrected iHep (Figure 3I; and Figure S4F, Supporting Information). The activities of respiratory chain complex V and I were reduced in patient iHep relative to control and corrected iHep (Figure 3J,K). For metabolites, we analyzed lactate/pyruvate ratio and observed a significant increase in patient iHep compared to control and corrected iHep (Figure 3L). Mitochondrial function is related to reactive oxygen species (ROS) level, so we detected ROS using a general oxidative stress indicator (H_2_DCFDA). We found a higher level of ROS in the patient iHep than control and corrected iHep (Figure 3M). All these results indicate that mitochondrial respiration activity is impaired in patient iHep.

### 2.4 Patient iHep‐Orgs and iHep Sensitivity to Iron Overload‐Induced Ferroptosis is Rescued by the GSH Precursor, NAC

Iron overload was regularly detected in hepatocytes of DGUOK mutant patients by liver histological examination.^[^ ^4^ ^]^ Thus, we checked the expression of seven genes (*TF, TFRC, DMT1, FPN, IRP1, IRP2, HAMP*) in iron metabolism in control, patient and corrected iHep and iHep‐Orgs. We showed that expression of transferrin (TF) increased in both patient iHep and iHep‐Orgs (Figure S5, Supporting Information). TF, a plasma iron carrier, expresses in liver and binds to plasma iron for transport.^[^ ^31^ ^]^

**Figure 4:**
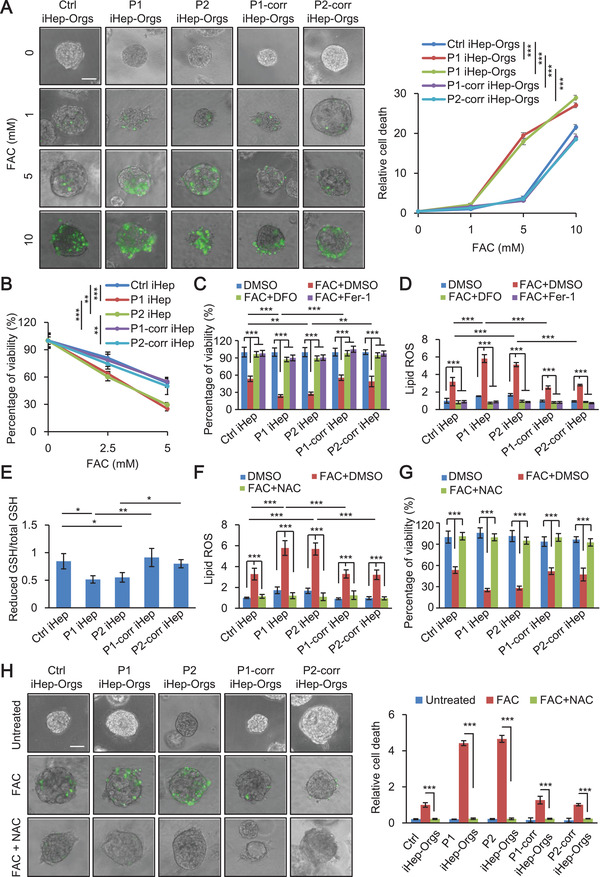
Patient iHep‐Orgs and iHep sensitivity to iron overload‐induced ferroptosis is rescued by the GSH precursor, NAC. A) Live‐cell imaging of control, patient and corrected iHep‐Orgs incubated with SYTOX Green with indicated concentration of FAC for 72 h. Scale bar, 50 µm (left). Quantification of relative cell death of control, patient, and corrected iHep‐Orgs (right) (*n* = 3, 50 organoids per group with three biological replicates). B) Cell viability of control, patient, and corrected iHep after treatment with FAC for 48 h. C) Measurement of cell viability of control, patient and corrected iHep with or without FAC and two ferroptosis specific inhibitors, DFO and Fer‐1 for 48 h (FAC, 5 × 10^−3^ m; DFO, 0.5 mµ; Fer‐1, 10 × 10^−6^ m). D) Measurement of lipid peroxidation in control, patient and corrected iHep with or without FAC and DFO, Fer‐1 for 24 h (FAC, 5 × 10^−3^ m; DFO, 0.5 mµ; Fer‐1, 10 × 10^−6^ m). E) Measurement of reduced GSH/total GSH in control, patient and corrected iHep. F) Measurement of lipid peroxidation in control, patient, and corrected iHep, after treatment with or without FAC and NAC for 24 h (FAC, 5 × 10^−3^ m; NAC, 5 × 10^−3^ m). G) Measurement of cell viability in control, patient and corrected iHep, after treatment with or without FAC and NAC for 48 h (FAC, 5 × 10^−3^ m; NAC, 5 × 10^−3^ m). H) Live‐cell imaging of control, patient, and corrected iHep‐Orgs incubated with SYTOX Green with FAC and NAC for 72 h (FAC, 5 × 10^−3^ m; NAC, 5 × 10^−3^ m). Scale bar, 50 µm (left). Quantification of relative cell death of control, patient and corrected iHep‐Orgs (right) (*n* = 3, 50 organoids per group with three biological replicates). These data are represented as mean ± SD (*n* = 3) and statistics was assessed using one‐way ANOVA A,B,E) and two‐way ANOVA C,D,F,G,H) followed by post‐hoc Holm–Sidak test, *p < 0.05;**p <0.01; ***p < 0.001.

To determine the pathological mechanism of iron overload and lethal liver failure in DGUOK mutant MDS, we used ferric ammonium citrate (FAC) to induce iron overload. iHep‐Orgs were treated with FAC at the indicated concentrations for 72 h, and a significant cell death was observed in patient iHep‐Orgs compared with control and corrected iHep‐Orgs at the concentration of 5 × 10^−3^ m FAC (**Figure** **4**A). We measured the intracellular iron by inductively‐coupled plasma mass spectrometry, and its concentration in organoids treated with 5 × 10^−3^ m FAC was around 78.8 µmol g^−1^. It has been reported that the degree of liver iron overload ranges from mild to severe,^[^ ^4^, ^32^ ^]^ and in a mild iron overload case report, the liver iron concentration was around 60 µmol g^−1^ (normal <36 µmol g^−1^).^[^ ^33^ ^]^ Thus, iron in our organoid model should be comparable to that in livers from patients. We also treated iHep with indicated concentrations of FAC for 48 h, and the similar result was observed that the percentage of surviving cells was less in patient iHep compared with control and DGUOK corrected iHep. (Figure 4B). These results indicate that both patient iHep‐Orgs and iHep were more sensitive to iron overload‐induced cell death. We then sought to determine which type of cell death is involved in the phenotype observed. We tried to rescue iron overload‐induced cell death using specific ferroptosis inhibitors deferoxamine (DFO) and ferrostatin‐1 (Fer‐1), apoptosis inhibitor ZVAD‐FMK or necroptosis inhibitor necrostatin‐1 in both iHep‐Orgs and iHep. Among the inhibitors used, only DFO and Fer‐1 could rescue iron overload‐induced cell death in both iHep‐Orgs and iHep (Figure 4C; and Figures S6 and S7, Supporting Information), indicating ferroptosis occurs upon FAC treatment. Besides FAC, we also tested other inducers of ferroptosis, erastin and RSL3. Erastin inhibits the activity of cystine/glutamate antiporter system Xc^−^, which causes GSH depletion. RSL3 directly inhibits GPX4.^[^ ^34^ ^]^ Both of them could induce a more severe ferroptosis in patient iHep (Figure S8, Supporting Information).

As lipid peroxidation plays a cardinal role in executing ferroptosis,^[^ ^9^ ^]^ we measured lipid peroxidation in control, patient and corrected iHep treated with FAC. We found that lipid peroxidation in patient iHep was higher than in control and corrected iHep. Additionally, this iron overload‐induced lipid peroxidation was significantly reversed by DFO and Fer‐1 (Figure 4D). As GPX4 is the only known enzyme responsible for reduction of lipid peroxidation,^[^ ^35^ ^]^ we analyzed the expression of GPX4 and activity of GPX. No significant differences were observed in control, patient and corrected iHep (Figure S9A,B, Supporting Information). We then examined GSH, which serves as a reducing co‐substrate for GPX4, and found that patient iHep displayed a reduction in GSH level compared with control and corrected iHep (Figure 4E). We also found that the reduction of GSH level could be recovered by NAC, a precursor of GSH (Figure S9C, Supporting Information). NAC decreased the lipid peroxidation caused by iron overload (Figure 4F) and alleviated the sensitivity of iron overload induced ferroptosis in patient both iHep‐Orgs and iHep (Figure 4G,H). These results indicate that the depletion of GSH in patient iHep causes an increase in lipid peroxidation and results in sensitivity to ferroptosis, which could rescued by the GSH precursor, NAC.

### 2.5 Ferroptosis of Patient iHep is Accompanied with Degradation of Ferritin in Lysosome

**Figure 5:**
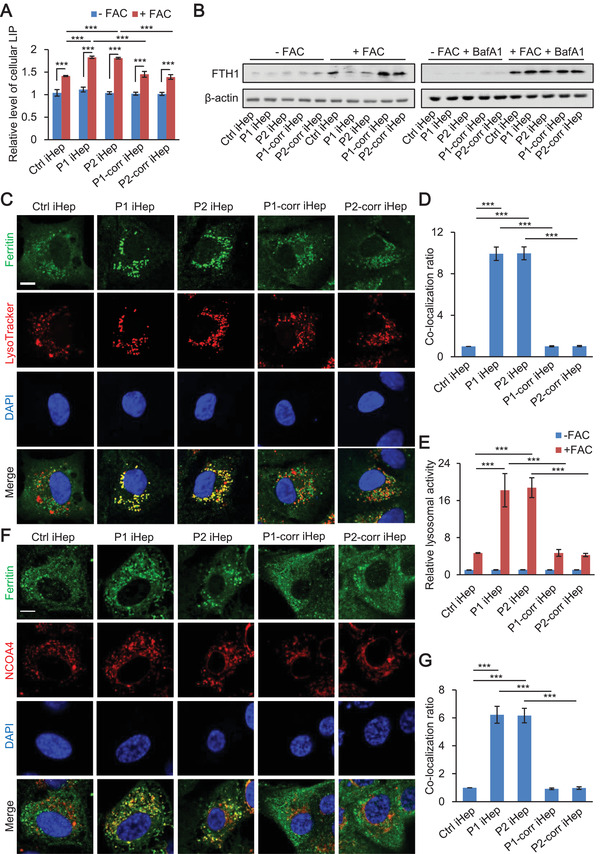
Ferroptosis of patient iHep is accompanied with degradation of ferritin in lysosome. A) Cellular free iron levels of control, patient and corrected iHep after treatment with or without 5 × 10^−3^ m FAC for 48 h. B) Western blotting of FTH1 in control, patient and corrected iHep after treatment with or without 5 × 10^−3^ m FAC for 48 h (left), with or without FAC for 48 h plus 50 × 10^−6^ m Baf A1 treatment (right). C,D) Representative confocal images of ferritin (green) and lysosomes (red) in control, patient, and corrected iHep after treatment with 5 × 10^−3^ m FAC for 48 h (left). Scale bar, 10 µm. The colocalization ratio of ferritin and lysosomes was shown in Pearson's correlation analyzed by image J in bar graph format from three independent experiments (right) (*n* = 3, 60 cells per group with three biological replicates). E) Relative lysosomal activity of control, patient and corrected iHep after treatment with or without 5 × 10^−3^ m FAC for 48 h (*n* = 3, 60 cells per group with three biological replicates). Representative images of this experiment are shown in Figure S10 (Supporting Information). F,G) Representative confocal images of ferritin (green) and NCOA4 (red) in control, patient, and corrected iHep after treatment with 5 × 10^−3^ m FAC for 48 h (left). Scale bar, 10 µm. The colocalization ratio of ferritin and NCOA4 was shown in Pearson's correlation analyzed by image J in bar graph format from three independent experiments (right) (*n* = 3, 60 cells per group with three biological replicates). These data are represented as mean ± SD (*n* = 3) and statistics was assessed using one‐way ANOVA D,G) and two‐way ANOVA A,E) followed by post‐hoc Holm–Sidak test, ***p < 0.001.

Previous studies have shown that cellular labile iron (LIP) released from ferritin degradation is required for lipid peroxidation upon ferroptosis.^[^ ^36^ ^]^ We measured LIP in iron overload iHep. We showed that LIP increased after treatment with 5 × 10^−3^ m FAC for 48 h and LIP level in patient iHep was higher than in control and corrected iHep (**Figure** **5**A). To determine whether the increased LIP is released from ferritin degradation, we performed western blot to examine the protein level of ferritin. Ferritin is a heteropolymer made of heavy and light ferritin chains (FTH1 and FTL). We showed that the expression of FTH1 increased in control and corrected iHep but not patient iHep after FAC treatment. Next, we treated the various iHep with FAC together with Bafilomycin A1 (BA), an inhibitor of lysosome acidification. In the presence of BA, the levels of FTH1 in patient iHep were similar to control and corrected cells, indicating that FTH1 is degraded in the lysosomes of patient iHep (Figure 5B).

To further determine the spatial controlling of ferritin degradation by lysosome, we analyzed the co‐localization of ferritin and lysosomes. Immunofluorescence showed substantial co‐localization of ferritin and lysosomes in patient iHep, much more than in control or corrected iHep (Figure 5C,D). We detected lysosome activity in control, patient and corrected iHep after treatment with or without FAC (Figure 5E; and Figure S10, Supporting Information). We showed lysosome activity in patient iHep was higher than control and corrected iHep after FAC treatment. As nuclear receptor coactivator 4 (NCOA4) has been reported to be involved in the degradation of ferritin,^[^ ^37^, ^38^ ^]^ we examined the colocalizations of ferritin and NCOA4 by immunofluorescence. The result showed that the colocalization ratio was higher in patient iHep than in control or corrected iHep (Figure 5F,G). Taken together, these results indicate that lysosomal degradation of ferritin is a step in ferroptosis of patient iHep.

### 2.6 Ferroptosis of Patient iHep is a Process with NCOA4‐Dependent Degradation of Ferritin in Lysosome

**Figure 6:**
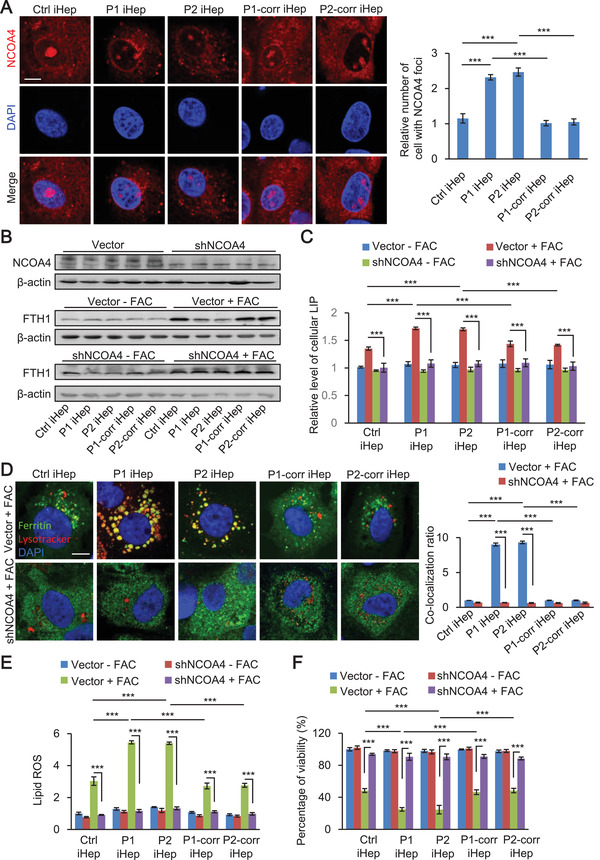
Ferroptosis of patient iHep is a process with NCOA4‐dependent degradation of ferritin in lysosome. A) Representative confocal images of NCOA4 (red) in control, patient and corrected iHep without FAC treatment (left). Scale bar, 10 µm. Quantification of cells displaying NCOA4 foci in control, patient, and corrected iHep (right) (*n* = 3, 60 cells per group with three biological replicates). B) NCOA4 knockdown rescued FTH1 degradation in patient iHep after treatment with 5 × 10^−3^ m FAC for 48 h. C) NCOA4 knockdown decreased cellular free iron levels of control, patient, and corrected iHep after treatment with 5 × 10^−3^ m FAC for 48 h. D) Representative confocal images of ferritin (green) and lysosomes (red) in control, patient, and corrected iHep expressing a control vector and shNCOA4 after treatment with 5 × 10^−3^ m FAC for 48 h. Scale bar, 10 µm. The colocalization ratio of ferritin and lysosomes was shown in Pearson's correlation analyzed by image J in bar graph format from three independent experiments (*n* = 3, 60 cells per group with three biological replicates). E) Measurement of lipid peroxidation in control, patient, and corrected iHep expressing a control vector and shNCOA4 after treatment with or without 5 × 10^−3^ m FAC. F) Measurement of cell viability of control, patient, and corrected iHep expressing a control vector and shNCOA4 after treatment with or without 5 × 10^−3^ m FAC. The data are represented as mean ± SD. Statistics were assessed using one‐way ANOVA, *p < 0.05; **p < 0.01; ***p < 0.001. These data are represented as mean ± SD (*n* = 3) and statistics was assessed using one‐way ANOVA A) and two‐way ANOVA C,D,E,F) followed by post‐hoc Holm–Sidak test, ***p < 0.001.

Next, we checked the immunofluorescence of NCOA4 in iHep before FAC treatment. Surprisingly, we observed that many NCOA4 foci appeared in patient iHep compared to control and corrected iHep (**Figure** **6**A). We then asked whether NCOA4 is a critical factor causing degradation of ferritin in ferroptosis of patient. We performed short hairpin RNA (shRNA) interference for silencing NCOA4 expression and observed an increase of FTH1 level in patient iHep during FAC induced ferroptosis (Figure 6B; and Figure S11, Supporting Information). Then, we measured the LIP level and found that NCOA4 knockdown decreased LIP level to an extent comparable with vector treatment iHep subjected to iron overload (Figure 6C).

To further determine the role of NCOA4 in lysosomal degradation of ferritin, we analyzed the co‐localization of ferritin and lysosomes upon NCOA4 knockdown. We observed that under NCOA4 deficiency ferritin showed a diffuse localization pattern without co‐localization with lysosome (Figure 6C,D). Furthermore, we found NCOA4 deficiency also blocked lipid peroxidation and ferroptosis in iron overload condition (Figure 6E,F). Together, these results indicate that NCOA4 mediates degradation of ferritin in lysosome upon iron overload‐ferroptosis induction.

## 3 Discussion

**Figure 7:**
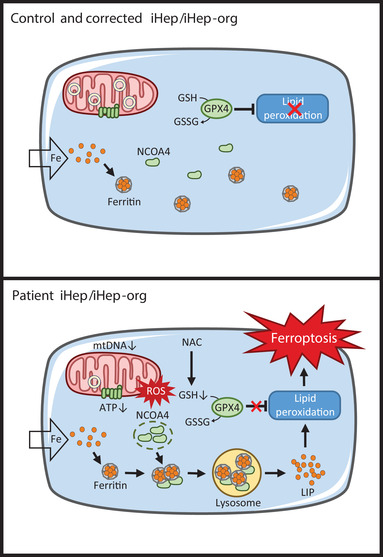
Model for ferroptosis of patient iHep and iHep‐Org. In control and corrected iHep/iHep‐Org, excess iron is packaged into ferritin in the cytoplasm to protect cells from reactive iron species. In patient iHep/iHep‐org, mtDNA depletion leads to mitochondrial dysfunction, reduced ATP production and ROS enhancement, which causes GSH exhaustion. Mechanistically, LIP released from ferritin which is degraded in lysosomes by a NCOA4‐dependent manner. Subsequent lipid peroxidation cause ferroptosis. This pathological ferroptosis process can be rescued by NAC.

In this study, we demonstrate the pathological mechanism underlying iron‐induced ferroptosis in DGUOK mutation patients, revealing the mystery of iron overload in clinical mtDNA depleted patient's hepatocytes. In patient hepatocytes, mtDNA depletion leads to mitochondrial dysfunction, reduced ATP production and ROS enhancement, which causes GSH exhaustion. Beside, more NCOA4 foci formate in patient iHep compared to control and corrected iHep. Then abundant cellular free iron is released from ferritin by lysosome degradation via a NCOA4‐dependent manner. The subsequent lipid peroxidation, eventually leads ferroptosis (**Figure** **7**). Moreover, without limitation to hereditary MDS, mtDNA depletion may occur in aging, degenerative diseases and other genetic diseases, all of which our findings shed light on.

We set up a combined iPSC model of 3D iHep‐Orgs and 2D iHep and isogenic controls by CRISPR/Cas9‐mediated gene correction for MDS diseases. Compared with 2D iHep, 3D iHep‐Orgs showed higher hepatic marker expression and stronger key liver functions. The other advantage of 3D iHep‐Orgs is that they can grow for multiple months in vitro and re‐enter cell cycle to undergo long term expansion.^[^ ^26^ ^]^ For the first time, we employed high purity hepatocyte‐organoids system to build human genetic liver disease model. The advance and advantage of this system is that we can focus on the impact of iron overloading on hepatocytes with mtDNA depletion and explore the mechanisms of hepatocyte death thoroughly, without cholangiocyte interference. Furthermore, using CRISPR/Cas9 gene editing, we generated DGUOK corrected, isogenic iPSCs which have normal karyotypes and can be generated to hepatocytes and liver organoids. It is a powerful strategy for modeling disease that ensures the consistency of genetic background, and it is much closer to the pathology of disease than gene knockout in normal people. It also provides the unique opportunity of both autologous hepatocytes for transplantation and personalized drug screening “in a dish”. Because DGUOK mutant MDS follows recessive inheritance, one allele correction is enough, which improves the practicality of gene‐editing as a therapeutic strategy. There are multiple studies have established the generation of live organoids from human hepatocytes, ESCs or PSCs, which have good 3D structure and liver functions, like albumin secretion, glycogen production, urea production and so on. Liver functions of 3D liver organoids still do not reach the levels of the primary human hepatocytes.^[^ ^26^, ^39^, ^40^, ^41^ ^]^ It is a great challenge to generate hepatic organoids with more mature hepatic phenotypic character in the field of organoids research. During our preparation of this manuscript, one report showed that NAD^+^ improves mitochondrial function in DGUOK‐knock out iPSC‐derived hepatocytes.^[^ ^42^ ^]^ This complementary work is consistent with our findings of mitochondrial dysfunction in DGUOK mutant patient hepatocytes. Our pure 3D hepatocyte‐organoids with gene correction system would be a good initiating example for iPSC modeling of other human genetic diseases.

NCOA4 has been known as a transcriptional coactivator of nuclear hormone receptors and locates in nucleus and cytoplasm.^[^ ^43^ ^]^ In our study, we showed that in control and corrected iHep, NCOA4 is almost dispersive in cytoplasm, while in patient iHep, NCOA4 formats foci. These distribution differences may be an alternative mechanism for mediating ferritin transport in lysosome to degrade. As ROS stress contribute to various cytosolic granules,^[^ ^44^ ^]^ ROS enhancement in patients may be involved in this NCOA4 foci formation.

Inspiringly, we found NAC may be promising as a potential therapeutic drug for DGUOK mutant patients. We demonstrated that NAC inhibits ferroptosis as a GSH precursor which can supply GSH for GPX4 to eliminate lipid peroxidation. As administration of NAC has been reported to be safe and beneficial in numerous clinical conditions, especially hepatic diseases,^[^ ^45^ ^]^ NAC holds great promise for therapies for MDS patients.

## 4 Conclusion

This study uncovers that mtDNA depletion and respiration dysfunction in the DGUOK mutant MDS patients iHep and both iHep and iHep‐Orgs of patients are more sensitive to iron overload‐induced ferroptosis which could recused by the glutathione precursor, NAC. Besides, this ferroptosis is a process mediated by NCOA4‐dependent degradation of ferritin in lysosome which can be rescued by NCOA4 silence. Our findings provide a foundation to investigate new therapeutic avenues for liver failure of MDS patient.

## 5 Experimental Section

##### Cell Culture, iPSCs Generation and CRISPR Editing

The fibroblasts were cultured in Dulbecco's modified Eagles medium (DMEM)/high glucose (HyClone) containing 10% fetal bovine serum (FBS) (Hyclone) with 1% nonessential amino acids (NEAA) (Gibco), 1% GlutaMAX (Gibco), 200 × 10^−6^ m uridine (Sigma), streptomycin (50 µg mL^−1^) and penicillin (50 U mL^−1^). Human fibroblasts were reprogrammed into iPSCs as previously described.^[^ ^27^ ^]^ Briefly, fibroblasts were infected with retroviruses encoding human SOX2, KLF4, OCT4, and c‐MYC (Addgene). After around 25 days, ESC‐like colonies were picked and cultured on Matrigel (BD) in mTeSR medium (STEMCELL Technologies).

Guide RNAs were designed to target DGUOK exon 6 (patient1) and intron 1 (patient2) using the Zhang lab CRISPR design tool (crispr.mit.edu). The annealed oligos which are complementary to each other containing the gRNA sequence were ligated to Cas9 plasmids pX330 which were already digested with BbsI by T4 DNA ligase (Life Technologies). Two ssODNs were designed for each patient as repair templates. One was the normal sequence, the other was the sequence containing synonymous mutations to avoid the DNA double‐strand continuously being cut. Guide RNAs and ssODNs sequences are listed in Table S1 (Supporting Information). 4 µg Cas9 and 2 µg ssODNs were transfected into DGUOK iPSCs using Nucleofector Kits for Human Stem Cells (Lonza). After transfection, cells were cultured in mTeSR1 with Rock inhibitor Y‐27632 (Selleck). After about 5 days, cells were plated in mTeSR1 with Y‐27632 at the density of 500 cells per 1 well of six‐well plate. After 2 weeks, individual colonies were expanded enough to identify the target sequence by PCR. Potential off‐target sequences for CRISPR/Cas9 are listed in Table S2 (Supporting Information).

##### Generation of iPSCs Derived Hepatocytes

The protocol of differentiation of iPSCs derived hepatocyte has been described previously.^[^ ^27^ ^]^ Activin A, BMP2, FGF4, HGF, KGF, and Oncostatin M were purchased from Peprotech. RPMI 1640, and B27 were purchased from Invitrogen. The iHep cells were maintained in hepatocyte culture media (HCM) (Lonza) within one week.

##### Generation of iHep‐Organoids

The protocol of generation of liver organoids has been described previously.^[^ ^26^ ^]^ We generated iHep‐organoids by seeding 50 000 iHep cells mixed with Matrigel into a well of a 24 well plate. After Matrigel solidification, 500 µL organoids generation medium was added per well. Organoids generation medium: AdDMEM/F12 (Thermo Scientific), 0.5% Penicillin‐Streptomycin, 1% GlutaMAX, 10 × 10^−3^ m HEPES, 1% B27 minus vitamin A, 15% R‐spodin1‐conditioned medium, 3 × 10^−6^ m ChIR99021 (Sigma), 10 × 10^−3^ m nicotinamide (Sigma),10 × 10^−9^ m gastrin (Sigma), 50 ng mL^−1^ EGF (Peprotech), 20 ng mL^−1^ TGF‐*α* (Peprotech), 100 ng mL^−1^ FGF7 (Peprotech), 100 ng mL^−1^ FGF‐10 (Peprotech), 50 ng mL^−1^ HGF (Peprotech), 2 × 10^−3^ m A83‐01(Tocris), 10 × 10^−6^ m Y‐27632, 1 × 10^−6^ m dexamethasone (Sigma), 10 ng mL^−1^ Oncostatin M. During generation, the medium was changed every 3 days. After 14 days generation, organoids were mechanically fragmented with pipette tips blowing and separated by centrifugation for 200 g 10 min. The pellets was re‐seed into Matrigel with a split ratio of 1:4 into a well of a 24 well plate. Then, the medium was changed every 3 days and the organoids could passaged every 10 days with a split ratio of 1:4.

##### Immunofluorescence

Cells were fixed with 4% paraformaldehyde (PFA) for 30 min, and permeabilized in 0.5% Triton X‐100 for 15 min. Then cells were blocked with 0.05% Triton X‐100 and 10% FBS for 30 min at room temperature. Then cells were incubated with primary antibodies for 2 h at room temperature or overnight at 4 °C. After washing, cells were incubated with corresponding secondary antibody for 1 h in dark. Then, cells were incubated with DAPI (Sigma) for 5 min in dark. Cells were imaged using Zeiss LSM 710 confocal microscope. The following primary antibodies were used: Anti‐DNA antibody (1:200, Merck Millipore, CBL186), TOMM20 antibody (1:200, Abcam, ab78547), ferritin antibody (1:200, Rockland, 200‐401‐090‐0100), NCOA4 antibody (1: 200, NOVUS, H00008031‐M05), SOX17 antibody (1:200, R&D, AF1924), AFP antibody (1:200, Proteintech, 14550‐1‐AP), ALB antibody (1:200, Abcam, ab207327). For the colocation detection of ferritin and lysosome, lysosome was stained by LysoTracker Deep Red (Invitrogen). Before fixed, cells were incubated with 0.1 × 10^−6^ m LysoTracker Deep Red for 1 h in cell incubator. Then, immunofluorescence of ferritin was executed as described above.

The protocol of immunofluorescence of organoids has been described previously.^[^ ^46^ ^]^ Before fixed, the organoids were extracted from Matrigel in ice‐cold recovery solution for 1 h. Then, organoids were fixed in 4% PFA in 4 °C for 1 h, then permeablilized in PBS supplemented with 0.1% Tween and blocked with 2% FBS for 1 h at room temperature. ALB and AFP antibodies were incubated at 4 °C overnight. After washing, cells were incubated with corresponding secondary antibody 4 °C overnight in dark. Then, cells were incubated with DAPI for 30 min in dark. Before imaged, organoids were cleared in a glycerol based clearing solution for 10 min. Organoids were imaged using Zeiss LSM 710 confocal microscope. 3D image analysis of organoids was performed using the Imaris software.

##### qPCR Analysis

Total mtDNA was extracted using a TIANamp Genomic DNA kit (TianGen). Total RNA was extracted according to the manufacturer's instruction. cDNA was synthesized by reverse transcription of 2 µg total RNA per sample using the ReverTra Ace kit (Toyobo). QPCR was performed using a CFX‐96 real‐time PCR detection system (BioRad) in conjunction with SsoAdvanced Universal SYBR Green Supermix (BioRad) using the following conditions: an initial denaturation step of 95 °C for 30 s, followed by 40 cycles of denaturation at 95 °C for 5 s and annealing‐elongation at 60 °C for 20 s. Primer sequences are listed in Table S3 (Supporting Information).

##### Western Blot

Cells were lysed in radio immunoprecipitation assay (RIPA) buffer (Beyotime) with protease inhibitor PMSF (Beyotime) and cocktail (Roche) on ice for 30 min. Protein concentrations were determined by BCA Protein Assay Kit (Sigma). Protein was separated by 15% polyacrylamide/sodium dodecyl sulfate polyacrylamide gel electrophoresi (SDS‐PAGE), and electro‐transferred onto polyvinylidene difluoride (PVDF) membrane (Merck Millipore). The membrane was blocked in 5% dry nonfat milk in Tris‐buffered saline plus 0.1% Tween 20 (TBST) for 2 h at room temperature, followed by incubation with primary antibody overnight at 4 °C. Secondary antibodies were goat antirabbit and goat antimouse with (horseradish peroxidase) HRP, which were applied in block for 1 h at room temperature followed by chemiluminescence imaging using the Electro‐Chemi‐Luminescence (ECL) (Merck Millipore). Relative ratio of respective density of each proteins band was quantified using Image J software. The following primary antibodies were used: MT‐ATP6 antibody (1:1000, Abcam, ab101908), MT‐ATP8 antibody (1:1000, SANTA CRUZ, sc‐84231), GPX4 antibody (1:1000, Abcam, ab125066), FTH1 antibody (1:1000, Cell Signaling, 3998), NCOA4 antibody (1: 200, NOVUS, H00008031‐M05), *β*‐actin antibody (1:2000, Sigma, A2066). The secondary antibodies were goat antirabbit with HRP (1:5000, Kangchen, KC‐RB035) and goat antimouse with HRP (1:5000, Kangchen, KC‐MM‐035).

##### Liver Function Assays

To assess glycogen storage, the organoids were stained by PAS staining kit (Polysciences), according to the manufacturer's protocol. LDL uptake was detected with DiI‐Ac‐LDL (Alpha Diagnostic). The organoids were treated with 10 µg mL^−1^ DiI‐Ac‐LDL for 2 h in cell incubator, then the organoids were extracted, fixed, incubated with DAPI and imaged by fluorescence microscopy as mentioned above. ICG uptake and release assay was performed by incubating 1 mg mL^−1^ ICG (Cayman) for 2 h in cell incubator. Images of ICG uptake were captured using a microscope, then the organoids were washed with PBS and refilled with organoids medium for 6 h in cell incubator. Then, images of ICG release were captured using a microscope. For albumin secretion detection, the supernatant of organoids was collected after medium changed for 48 h and determined using a Human Albumin ELISA kit (Bethyl Laboratory), according to the manufacturer's protocol. CYP3A4 activity was measured using a P450‐Glo Assay Kit (Promega), according to the manufacturer's protocol. In brief, the organoids were at first treated with 20 µM rifampicin for 48 h to induce CYP3A4 activity. Then the organoids were incubated with luminogenic substrate for 2 h and the supernatant was measured using a luminometer.

##### Measurement of ΔΨ_m_, OCR, ATP

Measurement of ΔΨ_m_ using Potentiometric dye TMRM (Invitrogen) has been described previously.^[^ ^47^ ^]^ Living cells were treated with 25 × 10^−9^ m TMRM in cell incubator for 30 min, and then replaced with 5 × 10^−9^ m TMRM for imaging using a Zeiss LSM 710 confocal microscope (Carl Zeiss) at 37 °C under 5% CO_2_. All image analysis was performed using the image J software.

Measurement of OCR using XF24 extracellular flux analyzer (Seahorse Biosciences) has been described previously.^[^ ^48^ ^]^ OCR was measured using the XF24 extracellular flux analyzer (Seahorse Biosciences). iHep were seeded on Matrigel‐coated XF24 plates at 50 000 cells per well allowed to attachment overnight. The next day, cells media were replaced with XF base assay medium supplemented with 25 × 10^−3^ m glucose, 1 × 10^−3^ m sodium pyruvate, and 2 × 10^−3^ m L‐glutamine in a non‐CO_2_ incubator for 1 h. OCR was were measured in XF media in response to oligomycin (1 × 10^−3^ m), carbonyl cyanide 4‐(trifluoromethoxy) phenylhydrazone (FCCP, 1 × 10^−6^ m) and rotenone (1 × 10^−6^ m) and antimycin A (1 × 10^−6^ m). All plotted values were normalized by counting cell number after measurement.

Measurement of ATP using using the ENLITEN ATP Assay System (Promega) has been described previously.^[^ ^47^ ^]^ Cell extraction was performed with 2.5% trichloroacetic acid, then neutralized and diluted in 10 × 10^−3^ m Tris‐acetate (pH 7.75). ATP levels were measured using the Luciferase/Luciferin reagent according to the manufacturer's protocol. For determination of mitochondrial ATP levels, cells were treated with 10 × 10^−6^ m oligomycin for 15 min before measurement.

##### Measurements of Lactate, Pyruvate, Complex I, and V Activity

Lactate and pyruvate were measured using the lactate assay kit and pyruvate assay kit (BioVision) according to the manufacturer's protocol. 1 × 10^6^ cells were collected then divided equally into 2 parts. One was for determination of lactate, and the other was for pyruvate. Mitochondrial complex I and complex V activity assays were determined using mitochondrial complex I activity assay kit (Abcam) and mitochondrial complex V (ATP synthase) activity assay kit (Novagen) following manufacturer's instruction.

##### Measurement of Cell Death and Cell Viability

For organoids, cell death was measured using SYTOX Green (Invitrogen) according to the manufacturer's instructions. In brief, organoids were stained 30 × 10^−9^ m SYTOX Green for 20 min and examined by a fluorescence microscope. The ratio of dead cells (SYTOX Green‐positive) to total cells was quantified using image J software.

For iHep, cell viability was measured using the cell counting kit‐8 (CCK‐8) viability assay (Beyotime) according to the manufacturer's instructions. Briefly, iHep were plated in a 96‐well plates and treated with various concentrations of FAC and drugs for determined times. The 10 µL CCK‐8 reagents were added to each well and incubated for 4 h. Then the plates were measured by a microplate reader (EPOCH2, BioTek Instruments) at 450 nm.

##### Flow Cytometry

Total ROS and lipid ROS were measured by flow analysis with H2DCFDA (Sigma) and C11‐BODIPY (581/591) (Invitrogen) staining according to the manufacturer's protocol, respectively. To analyze total ROS and lipid ROS, cells were stained with 25 × 10^−6^ m H2DCFDA and 2 × 10^−6^ m C11‐BODIPY(581/591) for 30 min at 37 °C, respectively, followed by using a flow cytometer (Accuri C6, BD Biosciences).

Measurement of cellular LIP using the fluorescent probe Phen Green SK (Invitrogen) has been described previously.^[^ ^49^ ^]^ Cells were incubated with 5 × 10^−6^ m of Phen Green SK for 15 min in cell incubator. Then, cells were harvested and analyzed with a flow cytometer.

##### Measurements of GSH Levels

Intracellular GSH levels were measured using Glutathione Colorimetric Assay Kit (Bivison) according to the manufacturer's protocol. 1 × 10^6^ cells were collected then divided equally into 2 parts. One was for determination of reduced form of GSH, and the other was for total GSH.

##### Measurements of Lysosomal Activity

Lysosomal activity was measured using LysoSensor Green DND‐189 (Thermo Fisher), as described previously. Briefly, cells were cultured with 1 × 10^−6^ m (final concentration) LysoSensor for 1 h in cell incubator, then washed with PBS and incubated in fresh hepatocyte medium for 30 min. The images were captured using a fluorescence microscopy. The relative lysosomal activity was quantified using Image J software.

##### RNAi and Chemicals

Lentiviral vectors encoding shRNA targeting human NCOA4 were produced by the co‐transfection of the lentiviral vector pLKO.1‐puro with packaging plasmids PMD2.G and PSPAX2 into 293T cells using polyfectine (PEI). 293T cells were grown in DMEM/high supplemented with 10% FBS, streptomycin (50 µg mL^−1^) and penicillin (50 U mL^−1^). Medium was changed 12 h after transfection and the supernatant was collected 48 h after transfection. The supernatant was filtered through a 0.45 µm filter and centrifuged at 50 000×g for 2.5 h. Then the viruses were used to infect iHep in the presence of polybrene (Sigma). shNCOA4 target sequences: 5’‐CCCAGGAAGTATTACTTAATT‐3’. FAC (Aladdin, A100170), DFO (MCE, HY‐D0903), Ferrostatin‐1 (Selleck, S7243), Z‐VAD‐FMK (Selleck, S7023), necrostatin‐1 (Selleck, S8037), NAC (Aladdin, G1418036), Baf A1 (Sigma, 19–148).

##### Statistical Analysis

All statistical analyses were performed using GraphPad Prism 6 software. The data used in this study are presented as mean ± S.D, from three independent experiments. P values were determined by one‐way ANOVA and two‐way ANOVA followed by post‐hoc Holm–Sidak test, as indicated in the figure legends. P values of less than 0.05 were considered as statistically significant. More detailed information has been provided in each figure legend.

For further methods, please see the Supporting Information.

## Conflict of Interest

The authors declare no conflict of interest.

## Author Contributions

X.L. conceived, designed and supervised the project. J.G. designed and carried out most experiments. J.G. carried out most data analysis. L.D. and X.H. participated in iHep‐Org generation, western blot experiments, and data analysis. G.X., L.Y., and F.B. participated in mitochondrial respiration experiments. S.L. supplied control iPSCs materials and helpful suggestions. H.S., M.G., and L.Z. participated in CRISPR editing. H.H. provided the 293T‐HA‐Rspon1‐Fc cells. X.L. and J.G. wrote the manuscript. Y.W. participated in manuscript edit. All authors commented on the manuscript and declared no conflicts of interest.

## Supporting information

Supporting Information

## Data Availability

Data available in article supplementary material.
