# Supplementary material for: A Combined Model of Human iPSC‐Derived Liver Organoids and Hepatocytes Reveals Ferroptosis in DGUOK Mutant mtDNA Depletion Syndrome
Source: Adv Sci (Weinh). 2021 Mar 8;8(10):2004680. doi: 10.1002/advs.202004680 (PMC8132052; doi:10.1002/advs.202004680)
Supplement: Supplementary file 1 — Supporting Information [file ADVS-8-2004680-s001.pdf]

© 2021 Wiley-VCH GmbH

ADVANCED SCIENCE
Open Access

Supporting Information

for *Adv. Sci.*, DOI: 10.1002/advs.202004680

A Combined Model of Human iPSC-derived liver Organoids and Hepatocytes Reveals Ferroptosis in DGUOK Mutant mtDNA Depletion Syndrome

Jingyi Guo, Lifan Duan, Xueying He, Shengbiao Li, Yi Wu, Ge Xiang, Feixiang Bao,

Liang Yang, Hongyan Shi, Mi Gao, Lingjun Zheng, Huili Hu, Xingguo Liu

Supporting Information

Supplementary figure legends

Figure S1. Generation and characterization of DGUOK iPSCs. (A) High expression of endogenous (endo) ESC transcription factors in selected patient iPSC clones. The values refer to donor patient fibroblasts; H1 ESCs are used as the positive control. (B) Silencing of the exogenous transgenes (Tg) in selected DGUOK iPSC clones. The values refer to DGUOK fibroblasts transfected with the exogenous factors. (C) Normal karyotype of DGUOK iPSC clones. (D) Detection of DGUOK mutations of two DGUOK patients.

Figure S2. Detection of off-target for CRISPR/Cas9-mediated-DGUOK correction. Detection of off-target sequences of two sgRNA. The possible off-target sequences were offered by http://crispr.mit.edu/.

Figure S3. Identification of hepatic differentiation of DGUOK iPSCs. (A) Immunofluorescence for the marker proteins at different stages of DGUOK hepatic differentiation. Endoderm: SOX17; Hepatic progenitors: AFP; Hepatocyte-like cells: ALB. Scale bar, 50 μm (top); Scale bar, 10 μm (bottom). (B) Patient iHep express liver-specific genes as detected by qPCR analysis. (C) Accumulation of glycogen was observed by PAS staining in patient iHep. Scale bar, 50 μm. (D) Analysis of basic hepatic function in patient iHep by observation of ICG uptake and release. Scale bar, 50 μm. (E) Quantitation of ALB secretion in control, patient

and corrected iHep-Org. These data are represented as mean ± SD (n = 3) and statistics was assessed using one-way ANOVA followed by post-hoc Holm–Sidak test.

Figure S4. Mitochondrial dysfunction in patient iHep. (A) Transcript levels of mtDNA in patient and corrected iHep. (B) Relative mean fluorescence intensity of MitoTracker Deep Red in control, patient and corrected iHep. (C) Western blot of TOMM20 in control, patient and corrected iHep. (D) Anti- TOMM20 immunofluorescence in control, patient and corrected iHep. Scale bar, 10 μm. (E) Basal respiration, maximal respiration and mitochondrial ATP production capacity in control, patient and corrected iHep obtained from Figure 2H. (F) Relative ATP level reduction by oligomycin in control, patient and corrected iHep obtained from Figure 2I. These data are represented as mean ± S.E.M (A) (n = 3) and ± SD (B,C,E,F) (n = 3) and statistics was assessed using student's t test (A) and one-way ANOVA (E,F) followed by post-hoc Holm–Sidak test, *$p < 0.05$, **$p < 0.01$; ***$p < 0.001$.

Figure S5. Expression of genes in iron metabolism. (A) The relative expression level of genes in iron metabolism in control, patient and corrected iHep. (B) The relative expression level of genes in iron metabolism in control, patient and corrected iHep-Org. These data are represented as mean ± SD (n = 3) and statistics was assessed using one-way ANOVA followed by post-hoc Holm–Sidak test, *$p < 0.05$, **$p < 0.01$.

Figure S6. Apoptosis inhibitor and necroptosis inhibitor failed to rescue iron overload–induced cell death in iHep and iHep-Org. (A) Live-cell imaging of control, patient and corrected iHep-Org incubated with SYTOX Green with or without 5mM FAC plus Z-VAD-FMK, necrostain-1 for 72 h. Scale bar, 50 μm. (B) Quantification of relative cell death of control, patient and corrected iHep-Org. (C) Measurement of cell viability of control, patient and corrected iHep treated with or without 5mM FAC plus Z-VAD-FMK, necrostain-1 for 72 h. These data are represented as mean ± SD (n = 3) and statistics was assessed using one-way ANOVA followed by post-hoc Holm–Sidak test.

Figure S7. Ferroptosis inhibitor rescued iron overload–induced cell death in iHep-Org. Live-cell imaging of control, patient and corrected iHep-Org incubated with SYTOX Green with or without 5 mM FAC plus DFO and Fer-1 for 72 h. Scale bar, 50 μm. Quantification of relative cell death of control, patient and corrected iHep-Org. These data are represented as mean ± SD (n = 3) and statistics was assessed using one-way ANOVA followed by post-hoc Holm–Sidak test, ***$p < 0.001$.

Figure S8. Ferroptosis induced by erastin and RSL3 in iHep. (A) Cell viability of control, patient and corrected iHep after treatment with erastin for 48 h. (B) Cell viability of control, patient and corrected iHep after treatment with RSL3 for 48 h. These data are represented as mean ± SD (n = 3) and statistics was assessed using one-way ANOVA followed by post-hoc Holm–Sidak test.

Figure S9. Measurement of GPX4 expression and activity, and mitochondrial ROS level. (A) Western blotting of GPX4 in control, patient and corrected iHep. (B) Measurement of GPX activity in control, patient and corrected iHep. (C) Measurement of reduced GSH/total GSH in control, patient and corrected iHep, after treatment of NAC for 24 h. These data are represented as mean ± SD (n = 3) and statistics was assessed using one-way ANOVA followed by post-hoc Holm–Sidak test.

Figure S10. Lysosome activity in iHep. Representative images of the experiment shown in Figure 5E. The activity of lysosomes was determined by the relative fluorescent intensities of LysoSensor Green DND-189.

Figure S11. Knockdown efficiency of shNCOA4 in iHep. RT-qPCR analysis for detecting the NCOA4 knockdown efficiency in control, patient and corrected iHep relative to vector control. The data are represented as mean ± SD (n = 3) and statistics was assessed using two-way ANOVA followed by post-hoc Holm–Sidak test.

Supplementary table 1. Guide RNAs and ssODN sequences for CRISPR/cas9

Name	Sequence
P1 Guide RNA 5’	CACCGTCTGATGAACATTCCAGTGC
P1 Guide RNA 3’	AAACGCACTGGAATGTTCATCAGAC
P2 Guide RNA 5’	CACCGCCTTCGATGGAGAGCCTTCG

P2 Guide RNA 3’	AAACCGAAGGCTCTCCATCGAAGGC
G183 ssODN	TCCTTCCCACCTCTCTCATGAGGTCTTCTTGTTTGGTTACTTCCTCAGAAAAATCATCATTGACATCCAACACCAGCACTGGAATGTTCATCAGAGCCTCAAAGTGGAGCCTATAAGAAGCGTGAACGTTTGGGGGACAGAAAAAAAGAA
P1 ssODN (samesense mutation)	TCCTTCCCACCTCTCTCATGAGGTCTTCTTGTTTGGTTACTTCCTCAGAAAAATCATCATTGACATCCAACACGAGCACTGGAATGTTCATCAGAGCCTCAAAGTGGAGCCTATAAGAAGCGTGAACGTTTGGGGGACAGAAAAAAAGAA
P2 ssODN	AGCACCCTTCAGTTCCATGGCCAAGAGCCCACTCGAGGGCGTTTCCTCCTCCAGAGGCCTGCACGCGGGGCGCGGGCCCCGAAGGCTCTCCATCGAAGGCAACATTGGTAAGGGCCGGAAAGCGGCTGCCAAGCCTTGGCCTCCGCCACG
P2 ssODN (samesense mutation)	AGCACCCTTCAGTTCCATGGCCAAGAGCCCACTCGAGGGCGTTTCCTCCTCCAGAGGCCTGCACGCGGGGCGCGGGCCCCGCAGACTCTCCATCGAAGGCAACATTGGTAAGGGCCGGAAAGCGGCTGCCAAGCCTTGGCCTCCGCCACG

Supplementary table 2. Potential off-target sequences for CRISPR/Cas9

P1 CRISPR/Cas9

UCSC gene	sequence
NM_001017424	TCTGACTAACATTCCACTTCTAG
NM_006459	AGTGTTTAACATTCCAGTGCAGG
NM_001846	TGTGATGAACAGTCCAGAGTTGG
NM_006901	TCTGATGACCTTTCCAGAGAGGG
NM_020151	TCTGCTGTAACTTCCAGTGCAAG
NM_001167929	TTTGTTGTTCATTCCAGTGCCAG
NM_000996	TGTGATGAAAATTACAGGGCGAG
NM_001114734	TCTGATAAGCATTCCAGTAAAAG
NM_000799	TCCCATGGACACTCCAGTGCCAG

P2 CRISPR/Cas9

UCSC gene	sequence
NM_032847	CCTTTGAATGAAAGCCTTCGCAG
NM_001204888	CCTCCGAGGGAGCGCCTTCCAGG
NM_001001396	CCTGCCCTGGAGAGCCTTGGCAG
NM_018238	CCTTCGATGGGGATCTTTCAGAG

UCSC gene	sequence
NM_144720	CCTTCGATGGAGAGCGCCTGCGG

Supplementary table 3. Primers for qPCR

Gene	Sequence
endo*OCT4*-FP	CCTCACTTCACTGCACTGTA
endo*OCT4*-RP	CAGGTTTTCTTTCCCTAGCT
endo*SOX2*-FP	CCCAGCAGACTTCACATGT
endo*SOX2*-RP	CCTCCCATTTCCCTCGTTTT
NANOG-FP	TGAACCTCAGCTACAAACAG
NANOG-RP	TGGTGGTAGGAAGAGTAAAG
REX1-FP	TCGCTGAGCTGAAACAAATG
REX1-RP	CCCTTCTTGAAGGTTTACAC
TgpMXs-FP	GGGTGGACCATCCTCTAGAC
TgOCT4-RP	CCAGGTCCGAGGATCAAC
TgSOX2-RP	GGGCTGTTTTTCTGGTTG
TgKLF4-RP	GGAAGTCGCTTCATGTGG
Tgc-MYC-RP	CCTCGTCGCAGTAGAAATAC
ALB-FP	GGTGTTGATTGCCTTTGCTC
ALB-RP	CCCTTCATCCCGAAGTTCAT
AFP-FP	ATTGGCAAAGCGAAGCTG
AFP-RP	GCTGTGGCTGCCATTTTT
AAT-FP	GTGGAAGAGCCTCAGAAT
AAT-RP	TTGGTGTAACGAACTAATAGC
AHR-FP	CTTGAAATCCGGACCAAAAA
AHR-RP	AAGCAGGCGTGCATTAGACT
RXRa-FP	AGAAGGTCTATGCGTCCTTGG
RXRa-RP	CAGGCATTTGAGCCCGATG
NTCP-FP	ACAATACATGCGCTATGTCATCA
NTCP-RP	GCCACACTGCACAAGAGAATG
HNF3a-FP	AAGGCATACGAACAGGCACTG
HNF3a-RP	TACACACCTTGGTAGTACGCC
HNF3b-FP	ACTACCCCGGCTACGGTTC
HNF3b-RP	AGGCCCGTTTTGTTCGTGA
MRP3-FP	ATTTGGAATCTAACATCGTGGCT
MRP3-RP	AGGATGCGGAACAGGCAAAG

FMO3-FP	ATGGTTATCCTTGGGACATGCT
FMO3-RP	CGTTAGGCTTTACGGACACAAT
FMO5-FP	AGGCTTGATTCAGCCCTTAGG
FMO5-RP	AGGCTTGATTCAGCCCTTAGG
COMT-FP	GAAGGGGACAGTGCTACTGG
COMT-RP	CAGGAACGATTGGTAGTGTGTG
NNMT-FP	GAGATCGTCGTCACTGACTACT
NNMT-RP	CACACACATAGGTCACCACTG
SULT1A1-FP	CGGCACTACCTGGGTAAGC
SULT1A1-RP	CACCCGCATGAAGATGGGAG
CYP3A4-FP	ATGAAAGAAAGTCGCCTCG
CYP3A4-RP	TGGTGCCTTATTGGGTAA
CYP3A5-FP	GCACCACCTACCTATGATGCC
CYP3A5-RP	AGAGCATAAGTTGGAATCACCAC
CYP2C8-FP	CTTGCCCGCATGGAGCTATT
CYP2C8-RP	GTGGCAGAGAAACAATCCCTT
CYP2C9-FP	GGACAGAGACGACAAGCACA
CYP2C9-RP	TGCCCTTGGGAATGAGATAG
CYP2C19-FP	CAACAACCCTCGGGACTTTA
CYP2C19-RP	GTCTCTGTCCCAGCTCCAAG
ACTIN-FP	GCTATCCCTGTACGCCTCTGG
ACTIN-RP	CGCTCGGTGAGGATCTTCAT
MT-*ND1*-FP	CCTAGGCCTCCTATTTATTC
MT-*ND1*-RP	GAATGATGGCTAGGGTGAC
MT-*ND2*-FP	CTACGCCTAATCTACTCCAC
MT-*ND2*-RP	CTTTGAAGGCTCTTGGTCTG
MT-*ND3*-FP	ACCACAACTCAACGGCTACA
MT-*ND3*-RP	TTGTAGGGCTCATGGTAGGG
MT-*ND4*-FP	GGACTCCACTTATGACTCCC
MT-*ND4*-RP	GGTTGAGAATGAGTGTGAGGC
MT-*ND4L*-FP	TCGCTCACACCTCATATCCTC
MT-*ND4L*-RP	AGGCGGCAAAGACTAGTATGG
MT-*ND5*-FP	CTATCACCACTCTGTTCGCAG
MT-*ND5*-RP	GTGGTTGGTTGATGCCGATTG
MT-*ND6*-FP	CTAAAACACTCACCAAGACC
MT-*ND6*-RP	GGAATGATGGTTGTCTTTGG
MT-*COX1*-FP	GATTTTTCGGTCACCCTGAAG
MT-*COX1*-RP	CTCAGACCATACCTATGTATC
MT-*COX2*-FP	CTATCCTGCCCGCCATCATC
MT-*COX2*-RP	GATTAGTCCGCCGTAGTCGG
MT-*COX3*-FP	CACATCCGTATTACTCGCATC
MT-*COX3*-RP	GAAGTACTCTGAGGCTTGTAG
MT-*Cytb*-FP	CAAACTAGGAGGCGTCCTTG

MT-*Cytb*-RP	CTGGTTGTCCTCCGATTCAG
MT-*12S*-FP	CACTACGAGCCACAGCTTAA
MT-*12S*-RP	TCAGGGTTTGCTGAAGATGG
MT-*16S*-FP	GGCATGCTCATAAGGAAAGG
MT-*16S*-RP	GGCCGTTAAACATGTGTCAC
MT-*ATP6*-FP	GCCCTAGCCCACTTCTTACC
MT-*ATP6*-RP	TTAAGGCGACAGCGATTTCT
MT-*ATP8*-FP	CCCACCATAATTACCCCCAT
MT-*ATP8*-RP	TTTTATGGGCTTTGGTGAGG
TF-FP	GTGTGCAGTGTCGGAGCAT
TF-RP	CATCGGATGGAATGACGCTTT
TFRC-FP	ACCATTGTCATATACCCGGTTCA
TFRC-RP	CAATAGCCCAAGTAGCCAATCAT
DMT1-FP	TGGAGATCATGGGGAGTCTG
DMT1-RP	AAGAAAACCTGGTCCGGTGAA
FPN-FP	CTACTTGGGGAGATCGGATGT
FPN-RP	CTGGGCCACTTTAAGTCTAGC
IRP1-FP	AACCCATTCGCACACCTTG
IRP1-RP	ATGGTAAGCGCCCATATCTTG
IRP2-FP	TCGATGTATCTAAACTTGGCACC
IRP2-RP	GCCATCACAATTTCGTACAGCAG
HAMP-FP	CTGACCAGTGGCTCTGTTTTC
HAMP-RP	GAAGTGGGTGTCTCGCCTC
NCOA4-FP	GAGGTGTAGTGATGCACGGAG
NCOA4-RP	GACGGCTTATGCAACTGTGAA

Supplementary methods

PAS staining

PAS staining for glycogen was performed using a PAS staining kit (Polysciences) according to the manufacturer’s protocol. In brief, iHeps in culture dishes were fixed in 4% PFA and were treated with 0.5% Periodic Acid, Schiff’s Reagent, 0.55% Potassium Metabisulfite, Acidified Harris Hematoxylin in turn as protocol described. Then cells was examined with a microscope.

ICG uptake and release

Prepare 100 mg/mL storage concentration of ICG (Cayman). Then, iHeps were treated with 1 mg/mL ICG for 1h in cell incubator. After washed 3 times with PBS, the cellular uptake of ICG was examined with a microscope. After examination, the dish was refilled with hepatocyte medium and was put in cell incubator for 6 h. Then, ICG elimination from cells was examined with a microscope.
